# Ferroptosis and Its Multifaceted Roles in Cerebral Stroke

**DOI:** 10.3389/fncel.2021.615372

**Published:** 2021-06-03

**Authors:** Yongfa Zhang, Xiaoyang Lu, Bai Tai, Weijia Li, Tao Li

**Affiliations:** ^1^Department of Neurosurgery, The First People’s Hospital of Yunnan Province, Kunhua Hospital, The Affiliated Hospital of Kunming University of Science and Technology, Kunming, China; ^2^Department of Neurosurgery, School of Medicine, The Second Affiliated Hospital, Zhejiang University, Hangzhou, China; ^3^Translational Neurosurgery and Neurobiology, University Hospital Aachen, RWTH Aachen University, Aachen, Germany

**Keywords:** ferroptosis, programmed cell death, iron overload, lipid peroxidation, ischemic stroke, hemorrhagic stroke, glutathione inhibition

## Abstract

Ferroptosis is a unique regulated cell death defined by the intracellular iron overload and distinct biological features compared with other well-known programmed cell death. Ferroptosis can be triggered by many causes including decreased expression of glutathione (GSH), inhibition of the function of glutathione-dependent peroxidase 4 (GPX4), and system x_c_^–^, all of which finally lead to the over-accumulation of lipid peroxides in the cell. Ferroptosis has been reported to play an important role in the pathophysiological process of various cancers. In recent years, much evidence also proved that ferroptosis is involved in the progress of cerebral stroke. In this review, we summarized the characteristics of ferroptosis and the potential relationship between ferroptosis and ischemic and hemorrhagic stroke, to provide new targets and ideas for the therapy of stroke.

## Introduction

Ferroptosis is a newly discovered iron-dependent regulated cell death, which has unique morphological and biochemical distinctions from other programmed cell death such as apoptosis, and necroptosis (Dixon et al., 2012). The characteristic morphological changes of ferroptosis are the shrunken mitochondria with a ruptured external membrane, reduced crista, a compressed internal membrane, and the intact cell nucleus (Dixon et al., 2012; Friedmann Angeli et al., 2014), while the apoptosis and necroptosis generally have swollen mitochondria and the broken nucleus (Dixon et al., 2012). Being different from the cell death caused by apoptosis due to activation of caspases and that caused by necroptosis due to activation of RIP1, RIP3, and MLKL (Kroemer et al., 2009; Galluzzi et al., 2012; Xie et al., 2016), the most essential biochemical attributes of ferroptosis are iron overload and the lethal accumulation of lipid peroxides and reactive oxygen species (ROS) within the cell (Stockwell et al., 2017), which can produce a large number of alkyl oxygen radicals, leading to fatal cell membrane damage and disorganization (Agmon et al., 2018). Ferroptosis has been proved to be closely related to a variety of diseases throughout the human body. For the central nervous system, it has been shown that ferroptosis plays an important role in many neurodegenerative diseases such as Alzheimer’s Disease (Cong et al., 2019; Masaldan et al., 2019), Parkinson’s Disease (Ayton et al., 2013, 2015), and Huntington’s Disease (Lee et al., 2011). Furthermore, in recent years, the potential roles of ferroptosis in the pathologic process of cerebral stroke are given with increasing focus (Zille et al., 2017; Karuppagounder et al., 2018). Thus, in this review, we intend to summarize the current knowledge and recent findings in the field of metabolic mechanisms of ferroptosis, its inducers and inhibitors, and the critical roles of ferroptosis in cerebral stroke pathophysiology.

## The Cellular Metabolic Mechanisms of Ferroptosis

**FIGURE 1:**
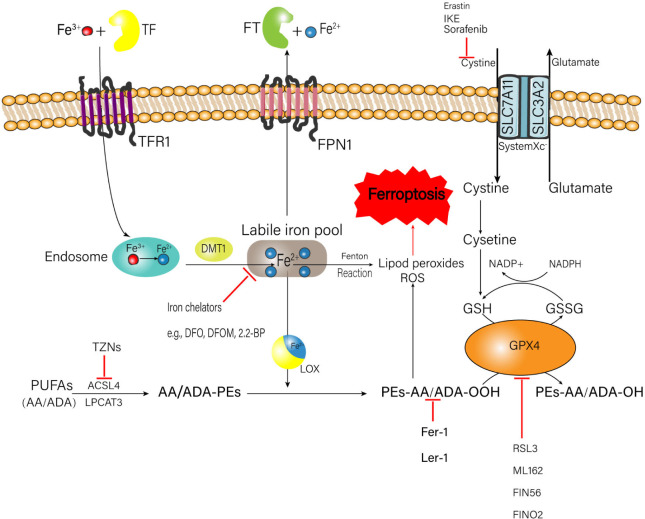
Metabolic processes of iron, amino acids, and lipid peroxide related to the happen of ferroptosis. A number of medicinal inducer have been shown to induce ferroptosis (e.g., erastin and RSL3). A variety of ferroptosis inhibitors also have been shown in the figure (e.g., Fer-1, lip-1, TZNs, and DFO). TF, transferrin; FT, ferritin; IKE, imidazole ketone erastin; TFR1, transferrin receptor 1; FPN1, ferroportin-1; SLC7A11, solute carrier family 7 member 11; SLC3A2, solute carrier family 3 member 2; DMT1, divalent metal transporter 1; ROS, reactive oxygen species; NADPH, nicotinamide adenine dinucleotide phosphate; GSH, glutathione; GSSG, oxidized glutathione; GPX4, glutathione peroxidase 4; DFO, deferoxamine; DFOM, deferoxamine mesylate; 2, 2-BP, 2, 2′-bipyridyl; PUFAs, polyunsaturated fatty acids; AA, arachidonic acid; ADA, adrenic acid; TZNs, thiazolidinediones; ACSL4, acyl-CoA synthetase long-chain family member 4; LPCAT3, lysophosphatidylcholine acyltransferase 3; LOX, lipoxygenase; Fer-1, ferrostatin-1; Lip-1, liproxstatin-1; RSL3, ras-selective lethal small molecules 3.

The occurrence of ferroptosis is associated with the metabolic processes of iron, amino acids, and lipid peroxide (Stockwell et al., 2017; Figure 1), which will be elaborated one by one as follows.

### Metabolism of Iron

Intracellular iron overload is a critical factor to trigger ferroptosis. Normally, the source of iron comes from two ways: (1) Food-derived iron including ferric iron (Fe^3+^) and ferrous iron (Fe^2+^); (2) Iron generated by the hemoglobin of senescent erythrocytes (Śliwińska et al., 2018). The absorbed Fe^2+^ is oxidized to Fe^3+^ in the small intestinal mucosa epithelial cells (Dong et al., 2008). Then the plasma Fe^3+^ is tightly bound to transferrin (TF) to form the complete TF, which can combine with the membrane protein transferrin receptor 1 (TFR1) to transport the Fe^3+^ into the endosome. After that, the Fe^3+^ is reduced to Fe^2+^ again by iron reductase (Hamaï and Mehrpour, 2017). Finally, the Fe^2+^ is transported into the labile iron pool in the cytoplasm through the divalent metal transporter 1 (DMT1), which is a commonly expressed protein with the ability to transfer a series of metal ions such as iron, crop, zinc, and cadmium (Garton et al., 2016) to perform their physio-pathology functions (Ji and Kosman, 2015; Knutson, 2019). Fe^2+^ can generate the ROS via the Fenton reaction, which is a type of reaction between ferrous iron and hydrogen peroxide (H_2_O_2_) referring to the creation of responsive varieties with the ability to oxidize a wide range of organic substrate (Winterbourn, 1995). In addition, Fe^2+^ is also an important component of the catalytic subunit of lipoxygenase (LOX), which can catalyze lipid peroxidation (Gaschler and Stockwell, 2017). Both mechanisms can trigger ferroptosis. Excessive Fe^2+^ is transported out of the cell through the ferroportin-1 (FPN1) on the cell membrane, to keep the intracellular Fe^2+^ concentration in a normal range. Then the Fe^2+^ outside the cell is stored in the ferritin (FT), to prevent the formation of hydroxyl radicals and ROS produced by H_2_O_2_ catalyzing (MacKenzie et al., 2008).

### Metabolism of Lipid Peroxide

Excess lipid peroxidation is another key factor to induce ferroptosis. Polyunsaturated fatty acids (PUFAs) are fatty acids with more than one double bond in their structure (Liu et al., 2015). PUFAs, especially arachidonic acid (AA) and adrenic acid (ADA) are very vulnerable to be oxidized, subsequently leading to the accumulation of lipid hydroperoxide (LOOH) and ROS (Das, 2019); the enrichment and location of PUFAs intracellularly will determine the degree of ferroptosis. Before the oxidation happens, PUFAs are esterified into membrane phospholipids, mainly of which is phosphatidylethanolamines (PEs) (Latunde-Dada, 2017). Then the PEs are further oxidized to phospholipid hydroperoxides (PEs-AA/ADA-OOH) by LOX, inducing ferroptosis. During the formation of AA/ADA-PEs, acyl-CoA synthetase long-chain family member 4 (ACSL4) and lysophosphatidylcholine acyltransferase 3 (LPCAT3), which serve as the promotors for the esterification of AA and ADA into PEs, are thought to play an important role (Yuan et al., 2016; Kagan et al., 2017). Doll et al. reported that targeted inhibition of ACSL4 can relieve cell and tissue damage introduced by ferroptosis (Doll et al., 2017). Similarly, inhibition of LPCAT3 can decrease the insertion of AA/ADA into membrane phospholipids. This evidence suggests that the restriction of ACSL4 and LPCAT3 may be a feasible healing strategy to stop ferroptosis-related illness.

### Metabolism of Glutathione and Amino Acid

Glutathione (GSH) is the major intercellular antioxidant against oxidative stress (Lu, 2013), which exerts an important duty on protecting cells from the ROS attack (Stockwell et al., 2017; Sun et al., 2018). It is suggested that cystine is the key to maintain the normal level of GSH (Imai et al., 2017). The system x_c_^–^ on the cell membrane consisting of a single-pass transmembrane regulatory protein solute carrier family 3 member 2 (SLC3A2) and a 12-pass transmembrane protein transporter solute carrier family 7 member 11 (SLC7A11) can exchange the extracellular cystine and the intracellular glutamate by a ratio of 1:1 simultaneously (Yang and Stockwell, 2016; Imai et al., 2017). When the cystine enters the cell, it is decomposed into cysteine, which combines with glutamate to form γ-glutamylcysteine under the catalysis of γ-glutamylcysteine synthetase (γ-GCS) (Fujii et al., 2019). Finally, the γ-glutamylcysteine combines with glycine to form GSH. The natural synthesis of GSH is the critical factor for glutathione peroxidase 4 (GPX4) to conduct its biological activity. GPX4 converts two molecules of GSH to oxidized glutathione (GSSG) (Maiorino et al., 2018) and reduces L-OOH to Phospholipids-H (L-OH) simultaneously, to prevent the accumulation of toxic lipid oxidation (Maiorino et al., 2018; Ursini and Maiorino, 2020). Selective ablation of GPX4 in neurons of mice can provoke lethal ferroptosis and neurological dysfunction (Chen et al., 2015). GSH also acts as the binding legend of Fe^2+^ in the labile iron pool to decrease the concentration of Fe^2+^, which can abate the production of Fe^2+^-related lipid peroxides and ROS.

## Inducers of Ferroptosis

As multiple mechanisms are involved in ferroptosis, according to different activated targets, ferroptosis inducers can be classified into three categories: (1) Inhibitors of GPX4, (2) Inhibitors of system x_c_^–^, (3) Small molecular inducers of iron loading.

### Inhibitors of GPX4

Ras-selective lethal small molecules 3 (RSL3) is found using phenotypic small molecule screens based on its ability to selectively facilitate oncogenic RAS mutant cell death (Dolma et al., 2003). RSL3 can bind to GPX4 by targeting an enzyme having a nucleophilic active site such as serine and cysteine, and inhibit the biological activity of GPX4 (Yang et al., 2014). ML162 is another small molecule compound that also can inhibit the activity of GPX4 (Dixon et al., 2015). In addition, both RSL3 and ML162 can enhance the expression of ACSL4 and LPCAT3 at the genetic level, which increases the accumulation of lipid peroxides (Dixon et al., 2015). FIN56 is a ferroptosis inhibitor acquired from CIL56, which can accelerate the degradation of GPX4 enzymatic function based on the chemical activity of acetyl-CoA carboxylase (Liang et al., 2019). FINO2, a course of organic peroxides that has many similar functions with artemisinins (Liang et al., 2019), is capable of indirectly inhibiting GPX4 and directly oxidate the Fe^2+^, resulting in an excessive build-up of lipid peroxides (Gaschler et al., 2018).

### Inhibitors of System x_c_^–^

Erastin is the classical ferroptosis inducers recognized by high-throughput screening of small-molecule libraries. Erastin could inactivate the function of SLC7A11 (xCT) in system x_c_^–^ to prevent cystine intake from GSH synthesizing and interfere with protein folding. Because of this, incompletely folded proteins gather in cells and cause cellular stress, thus giving rise to ferroptosis (Liang et al., 2019). The exact principle through which erastin hinders xCT remains unidentified. One possibility is that it inhibits xCT directly (Hirschhorn and Stockwell, 2019). Furthermore, erastin can combine with the voltage-dependent anion channel (VADC), a channel on the external membrane of mitochondria for controlling calcium ion exchange, bringing about mitochondrial impairment and further production of ROS by nicotinamide adenine dinucleotide phosphate (NADPH) dependent pathway (Yagoda et al., 2007). Imidazole ketone erastin (IKE) has a more powerful inhibiting effect and metabolic stability than erastin, which makes IKE an excellent choice for research of ferroptosis triggering *in vivo* and *in vitro* (Hirschhorn and Stockwell, 2019). L-α-aminoadipate, an amino acid intermediate in chain length between glutamate and cystine, is also an effective substrate inhibitor of system x_c_^–^ (Patel et al., 2004). The last common system x_c_^–^ inhibitor is sorafenib, which has been reported to trigger the ferroptosis of cancer cells by depleting the GSH (Lachaier et al., 2014). The RAF/MEK/ERK signaling pathway may exert an essential role in sorafenib inducing ferroptosis *in vitro* (Liu et al., 2006).

### Small Molecules Inducing Iron Loading

It has been shown that many small molecules such as ferrous chloride, ferrous ammonium sulfate, hemoglobin, and hemin trigger ferroptosis through iron overload, aggravating the Fenton reaction to generate more ROS, then promoting oxidation-related cell membrane damage.

## Inhibitors of Ferroptosis

With more and more studies focusing on ferroptosis, the possible duty of ferroptosis in diseases has been gradually revealed. To better study how to treat ferroptosis, lots of specific and effective ferroptosis inhibitors have been developed. According to the different inhibitory targets, inhibitors can be divided into (1) iron chelators, (2) lipid peroxidation inhibitors, and (3) ACSL4 inhibitors.

### Iron Chelators

The common iron chelators include deferoxamine (DFO), deferoxamine mesylate (DFOM), and 2, 2′-bipyridyl (2, 2-BP), among which DFO is the most widely used iron chelators approved by the Food and Drug Administration (FDA). It inhibits the acclamation of lipid peroxides by suppressing the Fenton reaction. DFO can effectively reduce stroke volume by increasing the expression of hypoxia-inducible factor 1 (HIF-1) (Baranova et al., 2007). It also has been reported that the DFO treatment can increase the resistance of neurons to impairment through regulating the microglial/macrophage heme oxygenase-1 (HO-1) expression in the SAH mouse model (LeBlanc et al., 2016). However, a randomized controlled trial regarding using DFO to treat intracerebral hemorrhage showed that there was no statistical difference in the clinical outcome after 90 days between the treatment group and the placebo group (Selim et al., 2019). Therefore, more studies are needed in the future to ascertain the role of DFO in the treatment of ferroptosis after stroke.

### Lipid Peroxidation Inhibitors

Lipid peroxidation inhibitors can be categorized into lipophilic radical-trapping antioxidants (RTAs) and LOX inhibitors according to their mechanism of action. RTAs are a class of molecules that is able to break the autoxidation of chain-propagating peroxyl radicals. The widely used RTAs are ferrostatin-1 (Fer-1) and liproxstatin-1 (Lip-1), which can inhibit lipid peroxidation linked to ferroptosis. Fer-1 can prevent erastin triggered-ferroptosis in HT1080 cells and is presently thought of as a probe for researching ferroptosis in various situations (Han et al., 2020). The activity of ferrostain-1 relies on the primary aromatic amine, which particularly hinders the accumulation of ROS from lipid oxidation (Xie et al., 2016). Lip-1 is a spiroquinoxalinamine by-product (Feng et al., 2019) and has a similar effect with Fer-1, but it only requires a lower dose to function due to the excellent absorption and distribution (Han et al., 2020). A-tocopherol, an analog of vitamin E (Ranard and Erdman, 2018), is also a potent type of RTAs to suppress ferroptosis *in vitro* (Angeli et al., 2017) and *in vivo* (Dalleau et al., 2013). It has been reported that A-tocopherol can inhibit breast cancer cell death induced by autophagy (Ma et al., 2017), which implies that ferroptosis may have a similar mechanism on cell death compared with autophagy. In addition, A-tocopherol can inhibit the function of 15-LOX, a subtype of LOX in the human body, by converting the non-heme iron of 15-LOX from the active Fe3+ state to the non-active Fe2+ state (Hinman et al., 2018). Another powerful kind of RTAs is tetrahydronaphthyridinols (TINs), of which the structure can significantly maintain the electron-rich phenols against autoxidation (Angeli et al., 2017). LOX inhibitors prevent ferroptosis through inhibiting the lipid peroxidation. LOXBlock-1 (LB1) is an inhibitor of 12/15-LOX, which is the dominant isoform of LOX in the brain (Singh and Rao, 2019). Karatas et al. (2018) reported that LB1 treatment decreased the infarct volume and the hemorrhage area in MCAO mice model. Similarly, Yigitkanli et al. (2013) found that LB1 could reduced infarct sizes and tissue plasminogen activator-associated hemorrhage in ischemia rat model. These studies imply that ferroptosis is closely related to the brain damage after stroke.

### ACSL4 Inhibitors

Acyl-CoA synthetase long-chain family member 4 is a critical enzyme to esterify AA and ADA, making it highly possible to be an inhibitory target for ferroptosis. Thiazolidinediones (TZNs), a class of drugs suggested for treating type 2 diabetes mellitus, have been shown to specifically suppress the ACSL4 activity and prevent ferroptosis (Kim et al., 2001; Doll et al., 2017). This effect may be a result of the chromanol ring, which endows tocopherols with antioxidant activity. Thus, TZNs provide a new perspective for pharmacological inhibition of lipid peroxidation and ferroptosis by suppressing the activity of ACSL4. Apart from that, rosiglitazone, one of the synthetic agonists for proliferator-activated receptor γ (PPARγ), is found to have the ability to selectively inhibit the activity of ACSL4 in cerebral ischemia rat models, thereby reducing neuronal lipid peroxidation and oxidative stress damage, and protecting neurological function (Sayan-Ozacmak et al., 2012).

## The Role of Ferroptosis in Cerebral Stroke

Cerebral stroke, including ischemic stroke and hemorrhagic stroke, is one of the most common causes of mortality and disability in modern society, which often results in devastating and irreversible brain impairment (Cordonnier et al., 2017; Steliga et al., 2020). Lots of research has proved that ferroptosis is closely allied to the pathophysiology of ischemic and hemorrhagic stroke and inhibition of ferroptosis can alleviate the secondary brain injury after stroke (Weiland et al., 2019; Chen et al., 2020), indicating that ferroptosis is a potential and critical therapeutic target for stroke.

### Ferroptosis in Ischemic Stroke

Ischemic stroke is the dominant subtype of stroke, defined by the unexpected cessation of cerebral blood flow (CBF) to an area of the brain, consequently producing a homologous loss of neurologic function. Ischemic stroke contains two important events: ischemia and reperfusion injury (IRI), the outcomes of which are able to prompt serious cellular impairment responsible for the poor prognosis of ischemic stroke (Yan et al., 2020). The specific signaling pathways and molecular mechanisms related to IRI are still not well recognized and are greatly disputed. A series of studies have reported that ferroptosis is involved in IRI in recent years (Xie et al., 2016; Doll and Conrad, 2017). First, iron accumulation has emerged after ischemia through inhibiting the expression of tau, which is a protein being able to form neuronal tangles in Parkinson’s disease and promote neuronal iron outflow (Tuo et al., 2017). Then the excess iron flows into the brain parenchyma through the interrupted BBB, boosting the generation of ROS via Fenton reaction, which promotes nucleic, proteomic, and membrane damage, ultimately triggering ferroptosis-related cell death (Liu et al., 2020). Moreover, ferroptotic cell death is found to be alleviated with the treatment of ferroptosis inhibitors Lip-1 and Fer-1 (Tuo et al., 2017). Apart from that, Guan et al. (2019) reported that carvacrol, a plentiful monoterpenic phenol in the essential oil of oregano and thyme, can rescue the IRI-induced hippocampal neuronal damage via suppressing ferroptosis by enhancing the expression of GPX4. Similarly, Lanet et al., found that the compound Chinese herbal medicine: Naotaifang, can also mitigate neuron ferroptosis after ischemic stroke via the TFR1/DMT1 and SCL7A11/GPX4 pathways (Lan et al., 2020). This research has highly proven that ferroptosis is closely linked to the pathology of ischemic stroke and may regulate the impairment after IRI.

### Ferroptosis in Hemorrhagic Stroke

Hemorrhagic stroke refers to an unexpected rupture of cerebral vessels that causes blood to flow into subarachnoid space, brain parenchyma, or ventricular system (Zhao et al., 2018). Therefore, a hemorrhagic stroke can be categorized into subarachnoid hemorrhage (SAH), intracerebral hemorrhage (ICH), and intraventricular hemorrhage (IVH). Compared with ischemic stroke, hemorrhagic stroke has a higher rate of mortality and morbidity due to severe neuronal death (Weiland et al., 2019). It has been shown that ferroptosis is involved in neuronal death after ICH *in vitro* and *in vivo* (Li et al., 2017; Karuppagounder et al., 2018; Zhang et al., 2018; Chen et al., 2019). Inhibition of the ferroptosis after ICH via ferrostatin-1 can alleviate neuronal death and enhance neurologic function (Li et al., 2017). Ferroptosis also plays an important role in early brain injury after SAH (Li et al., 2021). Cao et al. (2021) recently reported that the treatment with lip-1 protected HT22 cells against hemin-induced injury and reduced neurological deficits and neuroinflammation after SAH in mice. Additionally, earlier research has verified that the NF-E2-related factor 2 (Nrf2) pathway, a multifunctional signaling pathway that can control more than 250 genetics (Chang et al., 2014), is activated and provides neuroprotection after SAH (Chen et al., 2020). The expression level of Nrf2 is directly related to the occurrence of ferroptosis: an increase in Nrf2 expression inhibits ferroptosis; on the contrary, a decrease of it promotes ferroptosis (Fan et al., 2017). The mechanism by which Nrf2 inhibits ferroptosis involves two accepts: (1) Nrf2 upregulates the expression of xCT, thereby promoting the synthesis of GSH and GPX4 and enhancing the function of the antioxidant system (Fan et al., 2017); (2) Nrf2 promotes the expression of FT and FPN1 to store and export free iron, thereby reducing the accumulation of intracellular iron to prevent ferroptosis (Yang et al., 2017). Apart from that, Selenium is reported to be able to inhibit ferroptosis and protect neurons by promoting the expression of antioxidant GPX4 (Alim et al., 2019). These findings strongly suggest that ferroptosis aggravates the progress of hemorrhagic stroke and the inhibition of ferroptosis can reduce cell death and complications after a hemorrhagic stroke.

## The Possible Regulatory Mechanism of Ferroptosis in Stroke

Since the metabolisms of lipid peroxide, amino acids, and iron are closely related to ferroptosis, the regulatory mechanism of stroke ferroptosis may also be involved in these three aspects.

### Metabolism of Lipid Oxidation in Stroke

#### Inhibition of LOX Can Alleviate the Impairment Induced by Stroke

As we discussed above, LOX is the critical enzyme to catalyze PEs to form lipid peroxides. There are at least six subtypes of LOX in the human, among which the 12/15-LOX is a specific one due to its ability to directly oxidize lipid membranes containing PUFAs without the prior action of phospholipase and directly attack the mitochondria (van Leyen et al., 2014). It was reported that using the 12/15-LOX inhibitor can improve the neurological outcome and reduce the edema after ischemic stroke (Jin et al., 2008). This study provides evidence that 12/15-LOX is overexpressed and leads to both neuronal cell death and blood-brain barrier disruption after a stroke hit (Jin et al., 2008). Similarly, Gaberel et al. (2019) found that the expression of 12/15-LOX is upregulated in macrophages after SAH in mice, and inhibition of the 12/15-LOX pathway reduces EBI and protects the neurological function. In addition, 5-LOX, another subtype of LOX, is also found to have the ability to produce toxic lipids inducing ferroptosis. Treatment with N-acetylcysteine (NAC), a clinically approved thiol-containing redox modulatory compound that can inhibit the function of 5-LOX, is able to reverse brain injury and alleviate neuronal death (Karuppagounder et al., 2018). All the research indicates that LOX, especially 12/15-LOX is an innovative healing target to restrict brain injury after stroke.

#### ACSL4 Can Regulate Ferroptosis After Stroke

Acyl-CoA synthetase long-chain family member 4 has been found to be upregulated after ischemia and involved in ischemia-reperfusion injury (Gubern et al., 2013). This overexpression of ACSL4 is possibly controlled by miR-347, which is increased after ischemic stroke and upregulates ACSL4 at the transcriptional or post-transcriptional level (Gubern et al., 2013). Latunde-Dada et al., reported that inhibition of ACSL4 can alleviate ferroptosis and cell death in an ischemic rat model, and the special protein 1 (Sp1) is considered to be an essential transcription element for promoting the expression of ACSL4 via binding to the ACSL4 promoter region (Latunde-Dada, 2017; Li et al., 2019). However, studies related to this field remain limited. Thus in the future, more studies should be done to reveal the specific mechanism concerning the role of ACSL4 in stroke.

### Metabolism of Iron in Stroke

#### Iron Overload Leads to Ferroptosis After Stroke

Before the concept of ferroptosis is defined, people have already recognized that the accumulation of iron in the brain will contribute to secondary brain injury (Wu et al., 2003; Lou et al., 2009; Liu et al., 2019). Currently, iron overload is thought to be a critical cause to trigger ferroptosis after ischemic stroke owning to its role of increasing mitochondrial oxidative damage and infarct volume (Lipscomb et al., 1998; Chi et al., 2000; Castellanos et al., 2002). Additionally, a similar phenomenon is also observed in hemorrhagic stroke. For example, iron overload can induce mitochondrial damage in hippocampal neurons (Park et al., 2015) and increase significant perihematomal edema after ICH (Xi et al., 2006; Garton et al., 2016). The occurrence of iron overload after stroke is involved in two aspects: iron influx and iron efflux. The iron influx and efflux greatly depend on the iron metabolism-related proteins including TF, TFR1, DMT1, FT, and FPN1, as we mentioned above. These proteins are mostly controlled by the concentration of iron (Gill et al., 2018). Iron regulatory proteins (IRPs) are a class of proteins that can bind to iron-responsive elements (IREs) in specific mRNAs of iron metabolism-related proteins and control their usage. When the iron supply is decreased in the cell, IRPs will effectively bind to the IRE in mRNA of FT and FPN1, inhibiting the translation of FT and FPN1 (Eisenstein and Blemings, 1998). Conversely, when iron overload happens, the RNA binding function of IRPs is inactive in FT and FPN1 mRNA but active in TF mRNA, so the translation of FT and FPN1 is increased whereas the translation of TF is decreased, thereby preventing the iron import and prompting iron export (Eisenstein and Blemings, 1998; Silva and Faustino, 2015). However, studies found that the expression of TF is upregulated after stroke (Wu et al., 2003; DeGregorio-Rocasolano et al., 2018). This contradictory phenomenon indicates that there must be another undiscovered mechanism regulating TF. Apart from that, Hepcidin, a cysteine-rich polypeptide encoded by the human HAMP gene and secreted by the liver (Reichert et al., 2017), also has been identified as an important regulator of iron homeostasis recently (Almutairi et al., 2019). Hepcidin can bind to the iron efflux protein FPN1, thereby inhibiting its activity and promoting its degradation, finally suppressing the iron excretion (Gulec et al., 2014) and causing iron overload and oxidative stress damage in both ischemic and hemorrhagic stroke (Słomka et al., 2015; Tan et al., 2016; Xiong et al., 2016). It has been illustrated that iron overload in hepatocytes can activate the BMP6 signaling pathway and increase Hepcidin expression by regulating samd transcription factors (Ramos et al., 2011); inflammatory factors such as IL-6 can increase Hepcidin expression by activating the STAT3 signaling pathway (Wrighting and Andrews, 2006; Ding et al., 2011); hypoxia can inhibit Hepcidin expression by inducing erythropoiesis or secretion of PDGF-BB (Sonnweber et al., 2014). Furthermore, histone deacetylase (HDAC), a critical factor in regulating gene transcription closely related to cell proliferation and cell death (Gallinari et al., 2007), may play an important role in controlling the transcription of the HAMP gene. Drug screening experiments confirmed that HDAC inhibitors Pan have the potential to increase Hepcidin expression (Gaun et al., 2014), and *in vitro* studies also proved that hepatocytes could increase the expression of HAMP and Hepcidin (Mleczko-Sanecka et al., 2017). Therefore, it is highly possible that HDAC regulates iron overload and ferroptosis at the gene level of Hepcidin after stroke.

### Metabolism of Amino Acids in Stroke

#### GPX4 Inhibits Ferroptosis After Stroke

The expression of GPX4 is closely related to ferroptosis after stroke as it is able to inhibit lipid peroxidation. It has been shown that the upregulation of GPX4 can alleviate ferroptosis in both ischemic and hemorrhagic stroke. Alim et al. (2019) found that Selenium could promote the expression of antioxidant GPX4 to inhibit ferroptosis and protect neurons by activating the transcription factors TFAP2c and Sp1 in a hemorrhagic stroke rat model. Zhang et al. (2018) found that the genetic-upregulation of GPX4 efficiently relieved the secondary brain injury after ICH in the rat model including brain edema, blood-brain barrier leakage, and neuronal dysfunction. On the contrary, knocking out of GPX4 gene will aggravate brain injury (Zhang et al., 2018). Belayev et al. (2011) reported that Docosahexaenoic acid (DHA), an essential omega-3 fatty acid particularly abundant in nerve cell membrane phospholipids, may modulate the expression of the GPX4 gene via upregulating the Gpx4 Cytoplasmic Intron-sequence Retaining Transcripts (CIRT), which is an innovative Gpx4 splicing variant (Casañas-Sánchez et al., 2015). Overexpression of CIRT can harbor part of the initial intronic region, thereby increasing the capability of GPX4 to offer neuronal protection against oxidation based on the “sentinel RNA theory” (Casañas-Sánchez et al., 2015).

#### Overexpression of System x_c_^–^ Will Aggravate the Ferroptosis After Stroke

As is aforementioned, system x_c_^–^ serves as a glutamate/cysteine antiporter to produce GSH and GPX4, therefore it has a positive effect on inhibiting ferroptosis. The glutamate/cysteine exchange relies on the gradient of glutamate toward the cell rather than consuming adenosine triphosphate (ATP). When a stroke occurs, the concentration of extracellular glutamate will be escalated due to the lower glutamate uptake, increased exocytotic vesicular liberation, and non-vesicular glutamate release (Seki et al., 1999; Jabaudon et al., 2000), thereby leading to glutamate toxicity (Mou et al., 2019). As a result, the exchange will be blocked (Ignowski et al., 2018) and the production of GPX4 will be inhibited, finally initiating ferroptosis (Massie et al., 2015). The dysfunction of system x_c_^–^ may be triggered by the inactivation of its specific subunit xCT. However, the upregulation of system x_c_^–^ degree will result in greater glutamate release and excitotoxic impairment to oligodendrocytes (Pampliega et al., 2011). Similarly, astrocytic system x_c_^–^ can aggravate cerebral ischemic injury via regulating the deleterious and excitotoxic effects of IL-1β. Also, the upregulation of xCT can induce long-lasting glutamate excitotoxicity in an ischemic rat model (Hsieh et al., 2017). Taken together, these studies indicate that when the concentration of system x_c_^–^ reaches a certain threshold, it may aggravate the glutamate toxicity and ferroptosis rather than inhibit it. However, more studies are needed to confirm the specific role of system x_c_^–^ in stroke ferroptosis.

## Conclusion and Prospects

There is no doubt that ferroptosis plays a critical role in the progression and toxicity of ischemic stroke and hemorrhagic stroke, however, some confusing questions still need to be elucidated. First, although iron overload is thought to be the key factor to trigger ferroptosis, whether other metal ions are involved in ferroptosis remains unclear, as the latest research has revealed that copper and calcium may be also associated with the initiation of ferroptosis (Lewerenz et al., 2018; Maher et al., 2018; Weiland et al., 2019). Second, although people have known the important role of GPX and system x_c_^–^ in ferroptosis, the specific mechanism of regulating the change of GPX4 and system x_c_^–^ at the gene level is still not clear. Furthermore, while a series of studies have proved that ferroptosis can aggravate secondary brain injury after stroke, the underlying mechanisms and signaling pathways involved are still not understood. Finally, the relationship between ferroptosis, apoptosis, and autophagy remains unclear. It has been shown that ferroptosis, autophagy, and apoptosis will occur together in neuronal death after ICH via ultrastructural analysis (Li et al., 2018), however, whether ferroptosis can promote apoptosis or autophagy, or they can suppress each other after stroke requires to be studied in the years to come.

To recap, ferroptosis stands for a distinct type of programmed cell death, with a lot of its physiological functions yet to be specified. We believe that with the development of new biological technologies, more and more pathophysiological and physiological roles of ferroptosis will be gradually revealed.

## Author Contributions

YZ, XL, and BT manuscript writing. WL and TL manuscript revision and final approval of the manuscript. All authors contributed to the article and approved the submitted version.

## Conflict of Interest

The authors declare that the research was conducted in the absence of any commercial or financial relationships that could be construed as a potential conflict of interest.
